# Evaluating the Number of Trials for Stable Virtual Reality-Based Subjective Visual Vertical Measurement in Healthy Adults

**DOI:** 10.3390/jimaging12080373

**Published:** 2026-08-11

**Authors:** Tameto Naoi, Jun Watanabe, Keisuke Hamada, Mitsuya Morita

**Affiliations:** 1 Rehabilitation Center, Jichi Medical University Hospital, Shimotsuke 329-0498, Tochigi, Japanmorita@jichi.ac.jp (M.M.); 2 Department of Medicine, Division of Gastroenterology, Farncombe Family Digestive Health Research Institute, McMaster University, Hamilton, ON L8S 4K1, Canada; 3 Department of Surgery, Division of Gastroenterological, General and Transplant Surgery, Jichi Medical University, Shimotsuke 329-0498, Tochigi, Japan; 4 Division of Community and Family Medicine, Jichi Medical University, Shimotsuke 329-0498, Tochigi, Japan

**Keywords:** vestibular, SVV, VR, vertical, otolith, spatial orientation, test–retest reliability

## Abstract

Virtual reality-based subjective visual vertical (VR-SVV) has attracted attention as a potential solution to equipment-related limitations of conventional SVV testing. This study investigated the test–retest reliability and adequate trial number for stable assessment of vertical perception using VR-SVV in healthy adults. Participants performed 10 trials in a VR-SVV test and repeated the assessment after one week. SVV orientation and SVV variability were calculated. Test–retest reliability was evaluated using the intraclass correlation coefficient (ICC [1,2]), standard error of measurement (SEM), and minimal detectable change at the 95% confidence level (MDC_95_). The minimum number of trials required for stable assessment was examined by comparing results from fewer trials with those obtained from all 10 trials. SVV orientation and variability were −0.14° and 0.79°, respectively. ICC, SEM, and MDC_95_ were 0.62, 0.64°, and 1.76°, respectively. No adverse events occurred. SVV metrics derived from 6–9 trials differed by less than 10% from those obtained from 10 trials. VR-SVV is feasible and demonstrates moderate test–retest reliability. Six trials may be adequate for practical assessment of vertical perception.

## 1. Introduction

Recent advances in virtual reality (VR) technology have expanded its role as a visualization tool in rehabilitation medicine [1,2]. The subjective visual vertical (SVV) test assesses visuo-vestibular perception by requiring individuals to align a bar to the perceived vertical without external visual cues [3]. In this test, the perception of verticality is constructed based on internal models of vertical orientation, formed through the integration of multisensory graviceptive information, including somatosensory, visual, and vestibular inputs [4]. Recently, VR-based SVV (VR-SVV) has gained attention for its potential to overcome the limitations of conventional SVV tests, which require specialized equipment and/or a dark room. VR-SVV has already been used clinically, revealing impaired vertical perception associated with postural instability in patients with multiple sclerosis [5], Parkinson’s disease [6], vestibular migraine [7], and older adults with falls [8]. However, the methodological approaches vary widely in conventional SVV [3], and this variation may be even greater in VR-SVV owing to its portability and flexibility. In particular, no solid evidence exists regarding the number of trials required to assess SVV. Although 6–10 trials have been recommended, the number used in conventional SVV testing varies from 2 to 30 [3,9]. No such evidence has been reported for VR-SVV testing. Even in a static state, bodily perception is continuously fine-tuned through the integration of vestibular, somatosensory, and visual inputs, resulting in ongoing postural adjustments, while SVV orientation judgments are also influenced by psychological and cognitive states [3,4,9,10]. Therefore, SVV orientation may exhibit trial-to-trial variability. VR-SVV differs fundamentally from conventional SVV: a head-mounted display (HMD) presents separate images to each eye, which are integrated into a single visual percept [11,12], whereas conventional SVV uses a single screen [3]. To support broad applicability of VR-SVV, a reliable measurement procedure with an adequate number of trials is essential. This study examined the test–retest reliability of the VR-SVV test. The primary aim was to investigate the number of trials adequate for stable assessment of vertical perception.

## 2. Materials and Methods

### 2.1. VR-SVV System

**Figure 1:**
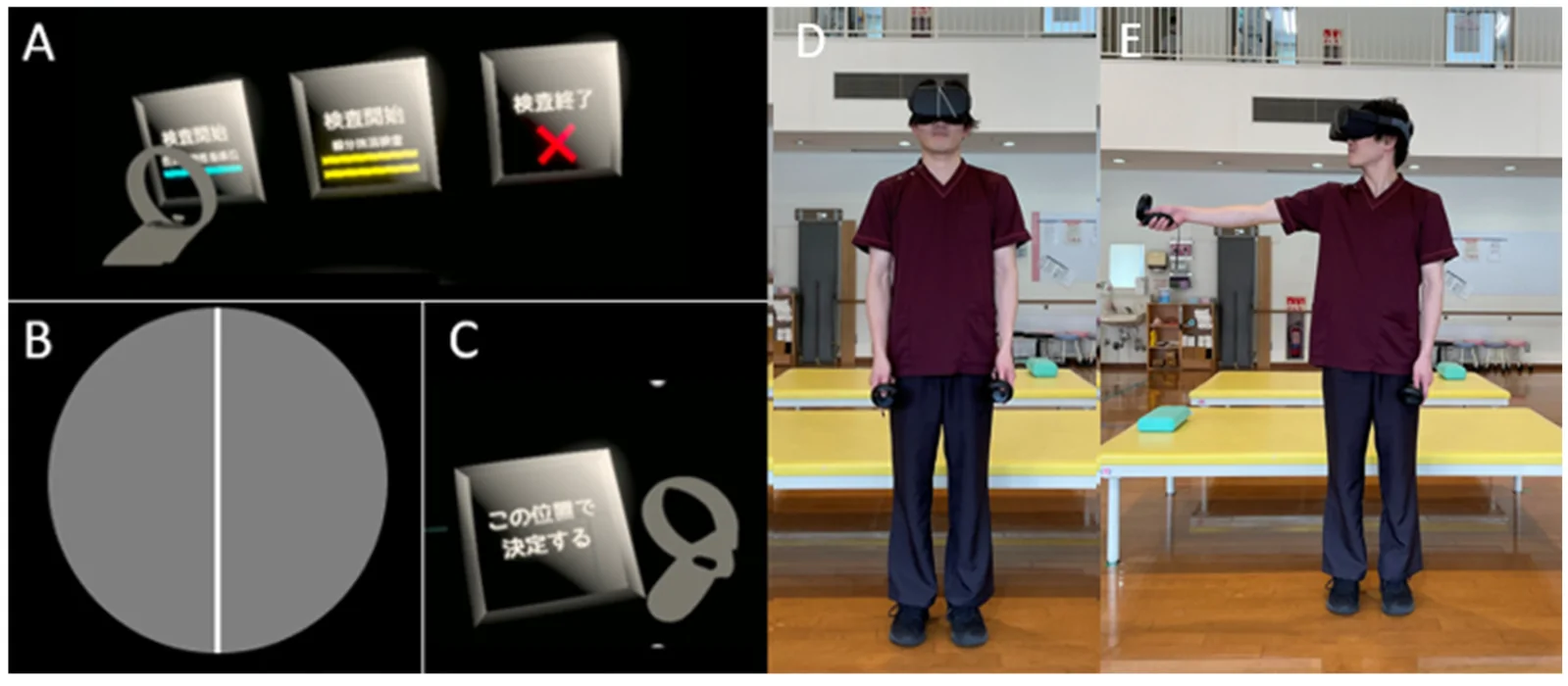
VR-SVV task procedure. (**A**) A participant selects the square button outlined in blue. The Japanese text accompanied by the blue and yellow lines indicates “Start the test”. The red cross also indicates the end of the test. (**B**) A white bar appears within a gray circle in the VR space. The participant rotates the bar using the controller. (**C**,**E**) The participant then turns their head to the right or left to locate the virtual confirmation button and presses it to confirm the SVV orientation. The Japanese text means “Confirm this position.” (**D**) The participant performs the VR-SVV test while standing.

In this single-center prospective observational study, the VR-SVV test was performed using an HMD, the first-generation Oculus Quest (Facebook Technologies, LLC., Menlo Park, CA, USA), which enabled the acquisition of SVV orientation data from participants. The Oculus Quest weighed 571 g and included a dual organic light-emitting diode display with a resolution of 1600 × 1440 per eye and a 72-Hz refresh rate. HMD stimuli were presented using an Oculus Quest program developed by Wide Soft Design Co., Ltd., Kobe, Japan. The VR-SVV system displayed a white bar within a gray circle against an all-black background without additional visual cues (Figure 1). Participants rotated the white bar using the Oculus Touch wireless controller and adjusted it to their perceived vertical. Participants then turned their head to the right or left to locate the virtual confirmation button and pressed it to confirm the SVV orientation. We confirmed that the head movement required to press the confirmation button had no appreciable effect on the SVV orientation. Clockwise and counterclockwise tilts were displayed as positive and negative values, respectively. The tilt of the bar was measured using an inclinometer in 0.1° increments, and the rotation speed of the bar was approximately 3.6°/s. The same HMD unit and VR-SVV application were used throughout data collection without any changes to the visual stimuli, measurement algorithm, or testing procedure.

### 2.2. Subjects

Because the VR-SVV system was custom-developed, this study aimed to establish its test–retest reliability and examine an appropriate number of trials in healthy adults. The inclusion criteria were age ≥18 years, normal or corrected-to-normal vision, no history of vestibular or neurological disorders, and full independence in activities of daily living. Exclusion criteria were as follows: current dizziness or headache, musculoskeletal limitations that prevented standing, and pregnancy. In addition, participants currently suffering from torticollis or trunk deformities such as scoliosis were excluded. Participants were recruited through advertisements at the Rehabilitation Center of Jichi Medical University Hospital between November 2020 and November 2025. Medical history was self-reported. A priori sample size calculation was performed for the intraclass correlation coefficient (ICC [1,2]) based on a one-way random-effects model for mean measurements. As a general reference, ICC values were interpreted as <0.50 (poor), 0.50–0.74 (moderate), 0.75–0.90 (good), and >0.90 (excellent) [13]. Assuming an expected ICC (good) of 0.75, a minimum acceptable ICC of 0.50 (moderate), an alpha level of 0.05, and a power of 0.80, the required sample size was estimated to be 45 participants. In this study, 48 participants were enrolled. Written informed consent was obtained from all participants.

### 2.3. Examination Protocol of VR-SVV

This study was conducted at the Rehabilitation Center of Jichi Medical University Hospital. After reading written instructions, participants wore the HMD and viewed the white bar binocularly in the VR environment. The examiner provided standardized verbal instructions to all participants before and during the tests. Before testing, all participants completed one practice trial to familiarize themselves with the SVV procedure; the result was not included in the analysis. In addition, the VR display was wirelessly mirrored and monitored on a laptop computer monitor to ensure that the participants operated the device properly. After adjusting the bar to their perceived verticality, participants were given a pause of several seconds to reconsider the bar orientation to reduce controller operation errors and judgment errors. The VR-SVV test was performed in an upright standing position with the head upright (Figure 1D). The examiner ensured that each participant maintained a natural upright head position without forced fixation. One week later, a retest was performed to assess test–retest reliability. This interval was selected to minimize immediate learning effects and recall of the previous bar orientations.

Each VR-SVV test consisted of 10 trials, and two outcomes were calculated: SVV orientation, defined as the mean value of the 10 trials, and SVV variability, defined as the standard deviation (SD) across the 10 trials. In this study, SVV orientation represented participant-level orientation bias, operationally defined as the signed deviation from the device-estimated vertical (0.0°). SVV variability reflects trial-to-trial fluctuations that may be influenced by variations in vertical perception, attention, cognition, decision-making, and potential device-related errors. The examiner remained nearby throughout the procedure to ensure safety. All assessments were performed by the same examiner (first author, TN). The entire sequence—from instruction to completion—required approximately 15 min. Participants were allowed to remove the HMD and discontinue the test if they experienced any unacceptable dizziness or discomfort. The vertical reference of the HMD was automatically determined using built-in inertial sensors. Therefore, before VR-SVV testing, the examiner performed the VR-SVV test and confirmed that the measured angle was within ±2.5° for three consecutive trials to detect any obvious miscalibration. A range of ±2.5° was used as a reference range for SVV orientation [3].

### 2.4. Statistical Analysis

Data were analyzed using the Statistical Package for the Social Sciences (SPSS, version 21; IBM Corp., Armonk, NY, USA). Sample size was estimated using EZR version 1.70 (Saitama Medical Center, Jichi Medical University, Saitama, Japan), a graphical user interface for R version 4.5.2 (R Foundation for Statistical Computing, Vienna, Austria), was used for the statistical analyses [14]. SVV orientation, SVV variability, and participants’ age were confirmed to follow a normal distribution using the Shapiro–Wilk test. Normally distributed data are presented as the mean ± SD. An independent-samples *t*-test was used to compare normally distributed continuous variables between male and female participants. Pearson correlation coefficients were used to examine the associations between age and SVV orientation and SVV variability. Statistical significance was set at *p* < 0.05. Test–retest reliability was evaluated using ICC. ICC reflects relative reliability. Absolute reliability was further evaluated using the Bland–Altman method to assess test–retest agreement and bias [15]. The 95% limits of agreement (LOA) were calculated as the mean difference ±1.96 × SD of the differences. Absolute error was assessed using the standard error of measurement (SEM) and minimal detectable change at the 95% confidence level (MDC_95_). SEM = SD × √(1 − ICC), MDC_95_ = SEM × 1.96 × √2 [16]. SEM represents the expected magnitude of measurement error. MDC_95_ represents the threshold above which an observed change is likely to exceed measurement error at the 95% confidence level. SVV orientation, SVV variability, ICC, SEM, and MDC_95_ were evaluated at each trial number from 1 to 10to investigate how these metrics changed with increasing numbers of trials. To examine measurement stability across different numbers of trials, each SVV metric was descriptively assessed based on its relative difference from the corresponding 10-trial estimate. We selected 10 trials as the reference because this number is practically feasible for a single SVV assessment while providing a sufficiently stable estimate for comparison with fewer trials. We defined the relative difference as $||{SVV\;metric}_{n}|-|{SVV\;metric}_{10}||/|{SVV\;metric}_{10}|\times 100(\%)$, where SVV metric_n_ and SVV metric_10_ represent each SVV metric calculated using n and 10 trials (n = 1–9), respectively; the SVV metrics included SVV orientation, SVV variability, ICC, SEM, and MDC_95_.

## 3. Results

A total of 48 healthy young adults (24 males and 24 females; mean age 30.6 ± 7.1 years; males, 31.3 ± 6.9 years; females, 30.0 ± 7.2 years) were enrolled in this study. Of these, 43 were right-handed and 5 were left-handed. All participants completed the VR-SVV test and retest without experiencing any adverse events, such as dizziness or falls. The median test–retest interval was 7 days (range: 7–8 days).

**Figure 2:**
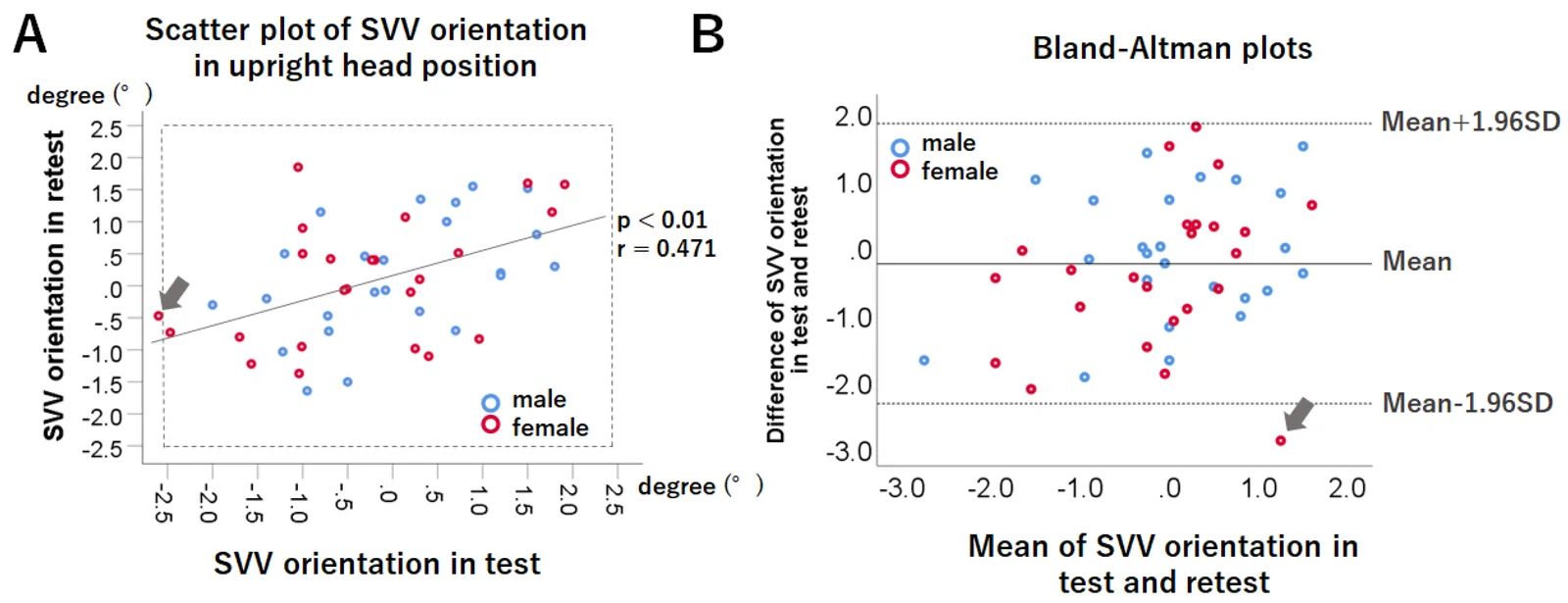
Test–retest results of VR-SVV in this study. (**A**) A scatter plot of SVV test and retest results. Forty-seven of 48 participants (97.9%) had SVV orientations within ±2.5° in both the test and retest. A statistically significant correlation was observed between test and retest (r = 0.471, *p* < 0.01). One female participant fell outside the reference range in the test (arrow). (**B**) A Bland–Altman plot for SVV orientation between test and retest. Forty-seven of 48 data points (97.9%) were within the LOA. One female participant fell outside the LOA (arrow). The solid horizontal line indicates the mean difference between test and retest measurements. The dotted lines represent the 95% LOA (mean ± 1.96 × SD). Abbreviations: LOA, limits of agreement; SD, standard deviation; SVV, subjective visual vertical.

SVV orientation was −0.14° (males 0.03°; females −0.31°, *p* = 0.31) during the test and 0.11° (males 0.15°; females 0.08°, *p* = 0.79) during the retest. SVV variability was 0.79° (males 0.70°; females 0.89°, *p* = 0.51) during the test and 0.69° (males 0.60°; females 0.78°, *p* = 0.79) during the retest. During the test, 47 of 48 participants (97.9%) showed SVV orientations within the reference range of ±2.5°, with 1 female participant recording −2.6° (Figure 2A, arrow). In the retest, all 48 participants (100%) demonstrated SVV orientations within ±2.5°. Bland–Altman analysis indicated that 47 of 48 differences (97.9%) fell within the LOA, with 1 female participant outside the LOA (Figure 2B, arrow).

**Figure 3:**
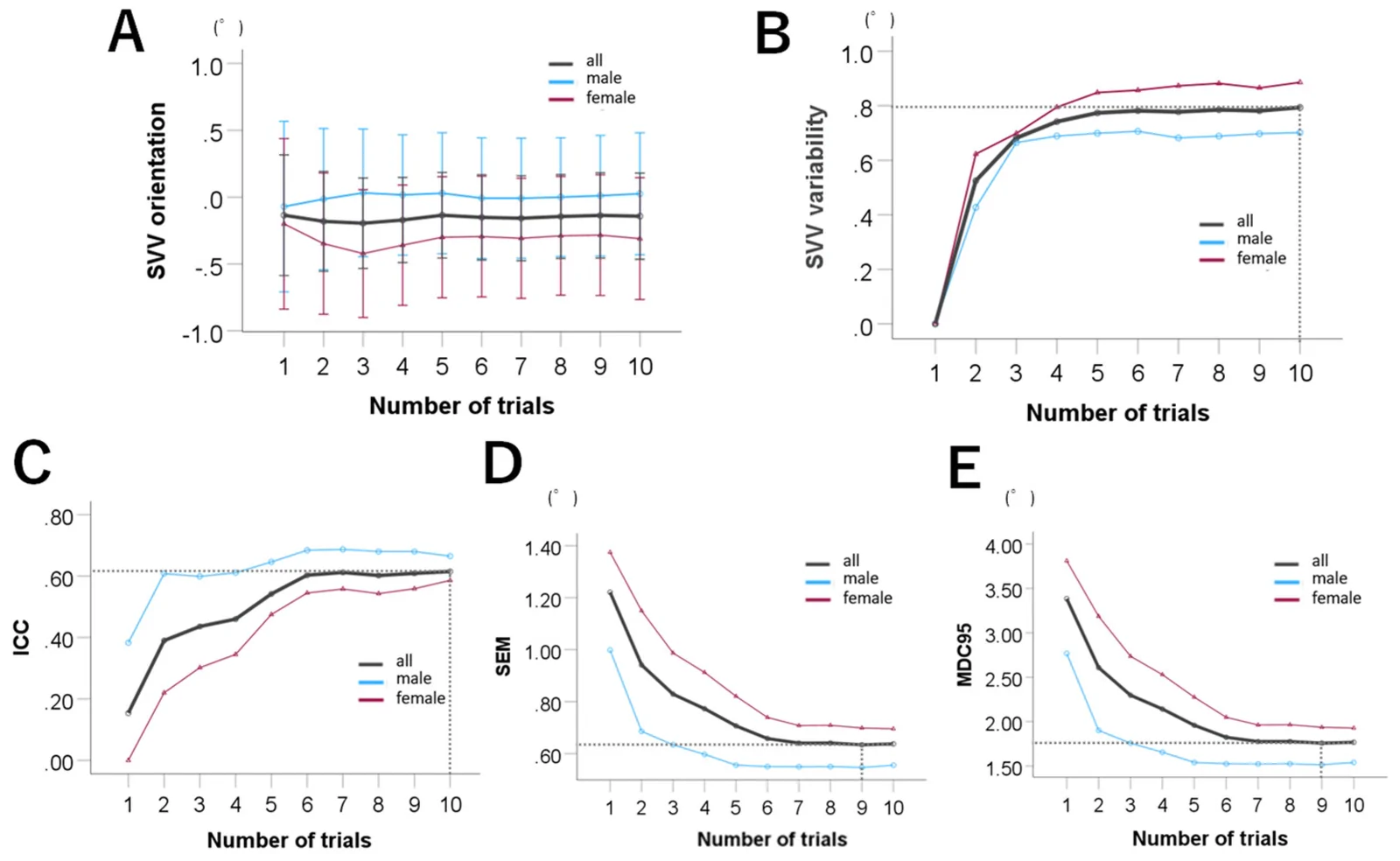
Changes in SVV metrics across repeated measurements from 1 to 10 trials. Data are shown for 48 participants (24 males and 24 females). (**A**) Changes in SVV orientation across trials. The SVV orientation remained within ±2.5° across trials. (**B**) Changes in SVV variability across trials. The horizontal and vertical dotted lines indicate the maximum SVV variability (0.79°) and 10 trials, respectively. (**C**) Changes in ICC across trials. The horizontal and vertical dotted lines indicate the maximum ICC (0.62) and 10 trials, respectively. (**D**) Changes in SEM across trials. The horizontal and vertical dotted lines indicate the minimum SEM (0.63°) and 9 trials, respectively. (**E**) Changes in MDC_95_ across trials. The horizontal and vertical dotted lines indicate the minimum MDC95 (1.76°) and 9 trials, respectively. SVV variability, ICC, SEM, and MDC_95_ progressively approached stable values as the number of trials increased, showing a plateau-like pattern. The black line indicates all participants. Blue and red lines represent males and females, respectively. Abbreviations: ICC, intraclass correlation coefficient; MDC_95_, minimal detectable change at the 95% confidence level; SEM, standard error of measurement; SVV, subjective visual vertical.

**Figure 4:**
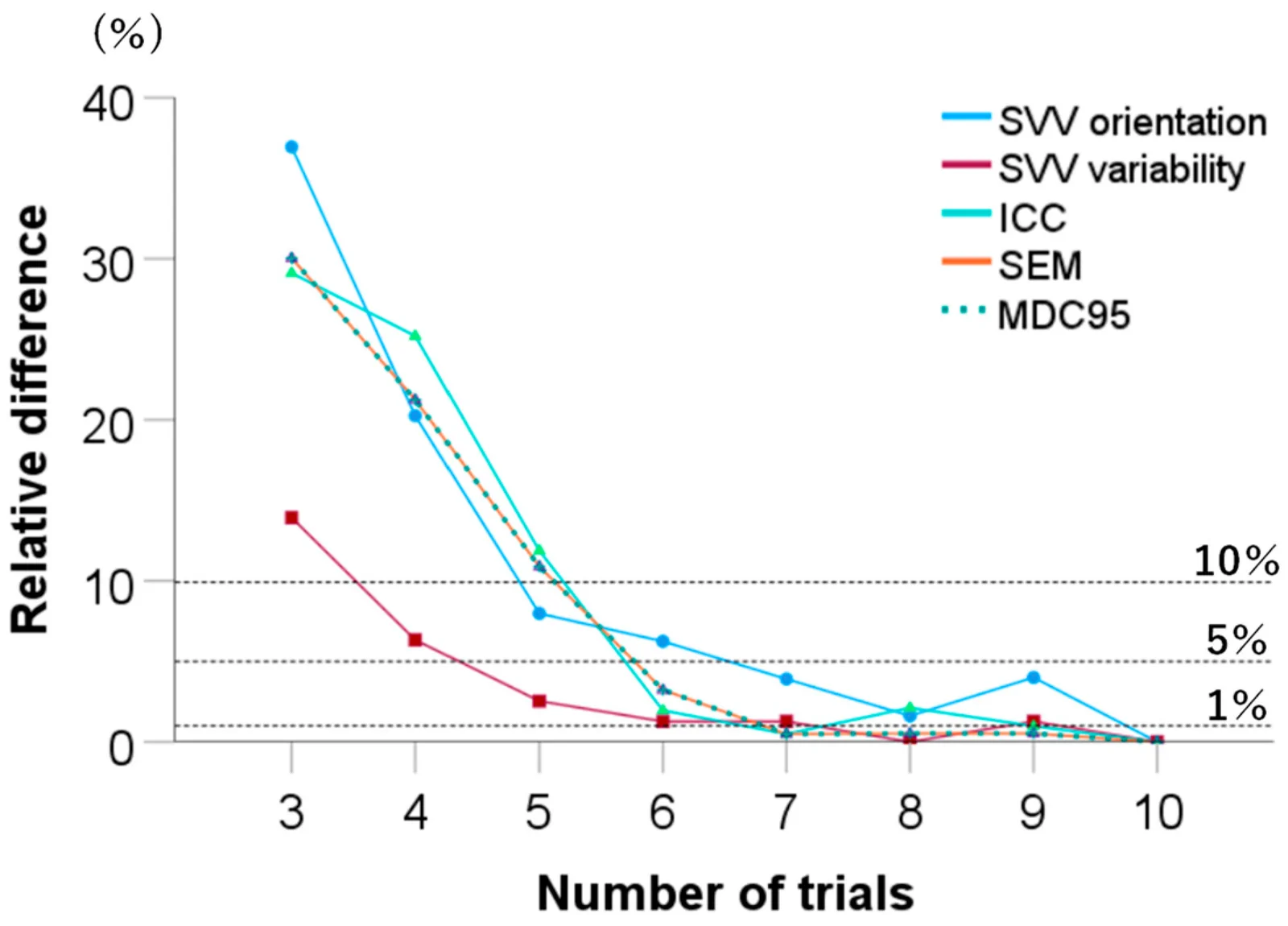
Changes in relative differences of SVV metrics across different numbers of trials. For better visualization of the smaller relative differences, only data from three trials onward are shown. All metrics had relative differences within 10% at six trials. From seven trials onward, all SVV metrics were within a 5% relative difference. SVV orientation continued to fluctuate from six to nine trials, whereas SEM and MDC_95_ converged toward their 10-trial estimates and were within a 1% relative difference from seven trials onward. The horizontal dotted lines indicate relative differences of 1%, 5%, and 10%. The 1%, 5%, and 10% reference lines represent descriptive benchmarks for visualizing convergence. Abbreviations: ICC, intraclass correlation coefficient; MDC_95_, minimal detectable change at the 95% confidence level; SEM, standard error of measurement; SVV, subjective visual vertical.

The mean bias between the test and the retest was −0.26°; no substantial systematic bias was observed. SVV orientation and SVV variability were not significantly associated with age. Test–retest reliability was moderate (overall ICC = 0.62; males, 0.67; females, 0.59) (Table 1). SEM was 0.64° (males, 0.55°; females, 0.69°) and MDC_95_ was 1.76° (males, 1.53°; females, 1.92°). Figure 3 shows the changes in SVV metrics across repeated measurements from 1 to 10 trials. SVV variability, ICC, SEM, and MDC_95_ changed markedly in the early trials and stabilized as the number of trials approached 10. Figure 4 shows that the relative difference in SVV orientation was 6.24%, whereas those in the other SVV metrics were within 5% at six trials. From seven trials onward, the relative differences in all SVV metrics were within 5%. In addition, linear regression analysis revealed no significant association between trial number and SVV orientation (β = 0.008, *p* = 0.866, R^2^ = 0.000), indicating no systematic drift.

## 4. Discussion

In this study, the VR-SVV test was safely performed without any adverse events in the upright standing posture. In the test and retest, 97.9% and 100% of participants, respectively, were within ±2.5°. Furthermore, 97.9% of the differences between paired measurements fell within the LOA in the Bland–Altman analysis. The ICC level was moderate. SVV orientation, variability, ICC, SEM, and MDC_95_ values stabilized as the number of trials approached 10. When SVV metrics were calculated using six or more trials, all SVV metrics except SVV orientation were within a 5% relative difference from the corresponding 10-trial estimates. In addition, females may show less stable VR-SVV measurements than males.

We first compared the reliability of our original VR-SVV system with that of previously reported VR-SVV systems. We identified 12 distinct VR-SVV systems: SVV app [17,18,19], Virtual SVV^TM^ [20,21,22], VIRVEST [5,8,23], Verti SVV [24], Virtualis Physio [25], ZT-VNG [26], Curator SVV [6], and five custom-developed systems built using VR application platforms such as Unity3D [7,27,28,29,30]. Despite methodological differences among studies, group-level SVV orientation was within the reference range of ±2.5° in all studies, including the present study [17,18,19,20,21,22,23,24,25,26,27,28,29,30]. At the individual level, however, not all participants fell within the reference range; previous studies reported that 90% (18/20) [25] and 86.7% (26/30) [29] of participants were within the ±2.5° range, consistent with the 97.9% observed in the present study. Similarly, Bland–Altman analyses in previous studies showed that 93.1–95.2% of the data points [17,18,24,28] were within the LOA, compared with 97.9% in the present study. SEM and MDC_95_ were reported to range from 0.90° to 1.39° and 1.76° to 3.85°, respectively [17,18,21,28]. Our SEM was lower than previously reported values, whereas our MDC_95_ was at the lower end of the previously reported range. Among previous studies, five VR-SVV systems across six studies were assessed for test–retest reliability using ICC, with reported ICC values ranging widely from 0.38 (poor) to 0.85 (excellent) [17,18,21,24,27,28]. Although the difference in ICC levels across studies has not been well discussed, ICC values can be influenced by system-specific characteristics, study protocols, and technical errors. In addition, the test–retest interval may be one reason for this variation; Ogihara et al. reported that the ICC over an interval of several weeks (0.38) was substantially lower than the within-day ICC (0.70) [17]. The ICC level in our study did not achieve a good level of reliability. However, ICC values may be lower in homogeneous samples with limited between-subject variability because ICC reflects the ratio of between-subject variance to total variance [31]. Small SEM and MDC_95_ values may coexist with a moderate ICC in a homogeneous sample. SVV values in this study were consistent with those previously reported, indicating that our VR-SVV system demonstrates feasibility comparable to previously reported VR-SVV systems.

To the best of our knowledge, only one study by Piscicelli et al. investigated the number of trials sufficient for SVV testing; Piscicelli et al. defined an ICC of ≥0.90 as high inter-trial reliability and considered six trials sufficient for stroke patients with SVV biases [9]. At the same time, the authors recommended 10 trials for non-selected patients with stroke because the ICC in patients with normal VV perception reached only 0.728 even with 10 trials [9]. Given the high fatigability and limited attentional resources in such patients, more than 10 trials may be impractical [32,33]. In this study, most SVV metrics had relative differences within 5% at six trials. Because no established thresholds are available for interpreting relative differences in SVV metrics, reference values of 1%, 5%, and 10% were used as descriptive benchmarks rather than as validated statistical or clinical thresholds. However, a relative difference within 5% is sufficiently small for the SVV test. Although no patients were included in this study, this finding is consistent with the 6–10 trials proposed by Piscicelli et al. [9]. The convergence to 10 trials may partly result from the statistical calculation itself; however, this finding may also indicate familiarization with VR-SVV testing, similar to that observed in conventional SVV testing. SVV orientation remained within ±2.5° across one to 10 trials (Figure 3A); however, SVV variability changed markedly in the early trials and stabilized as the number of trials approached 10 (Figure 3B–E). Excessive SVV variability can reduce confidence in the interpretation of SVV orientation itself. Winnick et al. classified SVV performance into four patterns based on accuracy and precision: accurate–precise, accurate–imprecise, inaccurate–precise, and inaccurate–imprecise [34]. SVV variability can reflect the robustness of the internal representation of verticality, enhanced by the congruency of somatosensory and vestibular signals [4,35] and the awareness of body orientation [28]. In previous VR-SVV studies, the number of trials per test varied from 2 to 10, and reported SVV variability ranged from 0.68° to 2.80° [17,18,19,20,21,22,23,24]. Differences in the number of trials may partly contribute to this wide range of reported values.

In exploratory analyses, female participants showed relatively greater SVV variability, SEM, and MDC_95_ values and lower ICC values than male participants (Figure 3B–E). The present study was not designed or powered to examine such differences. In fact, neither the present study nor previous VR-SVV studies have demonstrated significant statistical sex differences in SVV orientation or variability at the group level [19,20,25,30]. We consider that sex differences in VR-SVV results may be relatively small and have limited relevance at an individual level. Gerb et al. reported that dynamic SVV could detect sex differences that were not identified by conventional static SVV testing [36]. VR systems are often designed around the interpupillary distance of standard male users [37]. Therefore, women with narrower interpupillary distances may experience visual–vestibular mismatch [37]. Cybersickness also appears to be more common in women than in men [38]. In addition, visuospatial strategies used by women in a VR environment can differ from those of men, with women relying more on egocentric strategies and men more on allocentric strategies [39]. Vertical perception is a basic prerequisite for spatial orientation, and women have been reported to make less precise SVV judgments than men [37]. Yuan et al. differentiated between large- and small-scale spatial abilities and attributed these differences to gender-specific emotional processes in the parahippocampal gyrus and the adoption of egocentric spatial strategies [40]. The potential disadvantages experienced by women during VR-SVV testing may require further investigation.

This study has several limitations. First, the participants were healthy adults in their 20s to 40s, resulting in limited information on older populations and age-related effects. The finding that six trials may be sufficient for a single VR-SVV assessment should be considered preliminary and requires further validation in patients with vestibular disorders and older adults. Second, SVV variability should be considered when interpreting SVV orientation; however, its reference values have not yet been established. Third, no external calibration against a physical reference, such as a plumb line, spirit level, or calibrated inclinometer, was performed. In addition, the VR-SVV system was not compared with a conventional SVV test. Accordingly, the present study evaluated measurement reliability—that is, the consistency of repeated measurements—but did not establish measurement accuracy or criterion validity, which would require comparison with an external physical reference and an established SVV test, respectively. Therefore, systematic or mechanical measurement bias cannot be completely excluded. This remains an important methodological issue that should be addressed in future VR-SVV research. Fourth, because the values for each trial number were derived from the same 10 trials, the differences from the 10-trial estimates naturally decreased as the number of trials approached 10 (Figure 3 and Figure 4). This may have overestimated the apparent stability from six trials onward. However, the finding that the relative differences in most SVV metrics were within 5% from six trials onward provides practical support for the use of six trials. Lastly, the long-term consistency of VR-SVV systems remains uncertain because of potential hardware degradation and limitations in long-term software support.

Overall, our findings in healthy adults support the previously proposed range of 6–10 trials by Piscicelli et al. [9]. VR-SVV is promising for advancing our understanding of the mechanisms underlying postural control and gravity perception. Its interpretation also requires careful consideration of the inherent variability in vertical perception.

## Figures and Tables

**Table 1:** Results of the study; test–retest reliability values for SVV orientation and SVV variability.

	Mean (10 Trials)		ICC [1,2]	SEM	MDC_95_
	SVV Orientation (Test/Retest)	SVV Variability (Test/Retest)			
All subjects (n = 48)	−0.14°/0.11°	0.79°/0.69°	0.62 95% CI: 0.42–0.74	0.64°	1.76°
Male (n = 24)	0.03°/0.15°	0.70°/0.60°	0.67 95% CI: 0.24–0.85	0.55°	1.53°
Female (n = 24)	−0.31°/0.08°	0.89°/0.78°	0.59 95% CI: 0.06–0.82	0.69°	1.92°

ICC, intraclass correlation coefficient; MDC_95_, minimal detectable change at the 95% confidence level; SEM, standard error of measurement; SVV, subjective visual vertical; 95% CI = 95% confidence interval.

## Data Availability

The data presented in this study are available from the corresponding author upon reasonable request. The data are not publicly available due to ethical and privacy restrictions.

## Abbreviations

The following abbreviations are used in this manuscript:

CI/CIs	confidence interval(s)
EZR	Easy R
HMD	head-mounted display
ICC	intraclass correlation coefficient
LOA	limits of agreement
MDC_95_	minimal detectable change at the 95% confidence level
SD	standard deviation
SEM	standard error of measurement
SPSS	Statistical Package for the Social Sciences
SVV	subjective visual vertical
VR	virtual reality
3D	three-dimensional
